# Physicians’ and Residents’ Well-Being in Ecological System: A Scoping Review of Positive Deviance Strategies

**DOI:** 10.3390/healthcare13151856

**Published:** 2025-07-30

**Authors:** Hyoseon Choi, Janghee Park, Sanghee Yeo, Seung-Joo Na, Hyojin Kwon

**Affiliations:** 1 Department of Medical Education, Chosun University College of Medicine, Gwangju 61452, Republic of Korea; goodluck@chosun.ac.kr; 2 Education Research Institute, Seoul National University, Seoul 08826, Republic of Korea; 3 Department of Medical Education, Kangwon National University College of Medicine, Chuncheon 24341, Republic of Korea; jhpark22@kangwon.ac.kr; 4 Department of Medical Humanities and Medical Education, School of Medicine, Kyungpook National University, Kyungpook National University Hospital, Daegu 41405, Republic of Korea; shyeo@knu.ac.kr; 5 Department of Medical Education, CHA University School of Medicine, Pocheon 11160, Republic of Korea; sjna620@cha.ac.kr

**Keywords:** well-being, positive deviance approach, positive deviance, negative deviance, coping strategies, microsystem, macrosystem, mesosystem, doctor, residency

## Abstract

**Background/Objectives**: It is essential to explore and disseminate positive deviance strategies that promote resilience, mindfulness, and well-being beyond stress and burnout reduction strategies for residents and physicians who experience high levels of occupational stress. This scoping review maps studies that investigate positive deviance strategies to enhance the well-being of residents and physicians. **Methods**: A scoping review was conducted by PRISMA guidelines to identify English-language studies on strategies for physician well-being. PubMed, MEDLINE, Embase, and ERIC were searched using terms related to well-being, coping, and medical education. **Results**: Among the 38 studies included, 17 (44.7%) targeted physicians in graduate medical education (GME), while 19 (50%) focused on continuing medical education (CME). Positive deviance strategies were identified in 26 studies and were most frequently implemented at the microsystem level, such as small group interventions (e.g., coaching, mentoring, and workshops). These strategies addressed individual and organisational factors that contribute to physician well-being and were associated with improvements in life satisfaction, resilience, professional identity, and psychological safety. The review found that positive deviance strategies were often proactive, values-driven, and disseminated organically over time, emphasising the importance of longitudinal engagement and sustained institutional support. **Conclusions**: This scoping review highlights the growing use of positive deviance strategies, especially at the microsystem level, to promote physician well-being. These approaches emphasise sustainable, values-driven practices and may offer effective, context-sensitive solutions within healthcare systems.

## 1. Introduction

Physicians and medical residents are frequently subjected to considerable psychological burden, stress, and burnout, leading to a growing body of research aimed at addressing these issues. The mental health of medical professionals is critical not only to their quality of life and personal well-being but also to their ability to fulfil their professional roles and to ensure high-quality patient care [1,2].

Extensive international studies have sought to assess levels of stress and burnout among physicians [3,4], identify contributing factors [5,6], and examine the individual characteristics [7] that may render certain professionals more susceptible to psychological strain. While many studies have primarily focused on alleviating negative conditions such as stress or burnout, emerging evidence suggests that strategies promoting positive states—such as job satisfaction, enhanced quality of life, and well-being—may offer more stable and sustainable benefits over time [8].

Numerous studies have indicated that various factors influence physicians’ stress levels, well-being, and overall quality of life. These factors are commonly categorised into personal, organisational, and socio-cultural domains [5,9,10], and have also been examined through the lens of ecological systems [11,12]. Classifying determinants of physicians’ well-being within an ecological framework offers the advantage of facilitating the development of multi-layered and contextually nuanced strategies—encompassing interventions that target individual attributes, institutional structures, and broader socio-cultural and policy environments.

The positive deviance approach is a rigorously evidence-based, community-driven methodology that identifies and amplifies uncommon but successful behaviours or strategies practised by individuals or groups who, despite facing similar constraints and challenges as their peers, consistently achieve superior outcomes. Rather than imposing external solutions, this approach uncovers and disseminates contextually appropriate practices that already exist within the community, thereby fostering sustainable and scalable change [13,14,15,16]. Unlike traditional problem-solving methods that often seek external solutions, positive deviance uncovers effective strategies already present within communities. This approach has demonstrated success in various public health domains, such as improving hand hygiene and child nutrition, by identifying context-specific solutions that can be disseminated to achieve broader impact [17,18,19].

In healthcare delivery, the positive deviance framework has been used to address complex challenges, including patient safety [19]. For example, Lawton et al. distinguished between negative deviance—addressing unprofessional conduct and system failures—and positive deviance, which recognises individuals or teams who achieve exceptional outcomes through innovative and effective means. When applied to physician well-being, negative deviance strategies focus on mitigating stress and burnout. In contrast, positive deviance strategies aim to emulate practices that foster resilience, mindfulness, and overall well-being.

Recent research has increasingly adopted the positive deviance approach to identify and review effective strategies for complex healthcare problems. A systematic review categorised positive deviance strategies for improving vaccination coverage rates into structure, process, and context domains, offering a comprehensive framework for operationalising positive deviance in public health initiatives [20]. Similarly, researchers synthesised evidence on the use of positive deviance to enhance health service delivery and quality of care, highlighting its versatility and potential for quality improvement across diverse healthcare settings [17]. Additional systematic reviews have examined how healthcare organisations implement positive deviance strategies, providing insights into the processes and outcomes associated with this approach [18].

Collectively, these studies underscore the value of the positive deviance approach in uncovering, structuring, and disseminating effective solutions that already exist within healthcare communities. By doing so, positive deviance facilitates sustainable improvements in practice and outcomes, reinforcing its significance as a transformative strategy in healthcare systems.

Despite extensive research on physician stress and burnout, a significant gap remains in understanding and systematically mapping strategies that promote the positive aspects of physician and resident well-being, such as resilience, mindfulness, and professional identity, particularly through the lens of positive deviance within ecological systems. Most existing studies have focused on negative approaches aimed at mitigating burnout and stress. At the same time, the proactive, strengths-based positive deviance strategies have not been comprehensively reviewed or synthesised across different educational levels and ecological contexts. Furthermore, the characteristics and effectiveness of these positive deviance strategies at various system levels—from individual to macrosystem—are not well delineated in the literature. To address these gaps, this study poses the following research questions:

1. What are the prevailing trends in academic literature concerning strategies to enhance physicians’ professional well-being?
2. What positive and negative deviance strategies have been identified for promoting physicians’ well-being?
3. What are the defining characteristics of positive deviance strategies as applied to physicians’ professional well-being across ecological system levels?

Building on this, the primary purpose of this scoping review is to systematically map and synthesise existing studies on positive deviance strategies aimed at enhancing the well-being of residents and physicians, thereby providing a comprehensive understanding that can inform future interventions and policy development.

This scoping review primarily aims to examine the research landscape concerning positive deviance strategies intended to promote physicians’ well-being and quality of life. To provide a more comprehensive understanding of these strategies, the review also explores literature addressing negative deviance approaches as a point of contrast. Through this focus, the study seeks to generate meaningful insights into the development of effective and sustainable strategies to support physician well-being across healthcare systems.

## 2. Methods

### 2.1. Study Design

**Figure 1:**
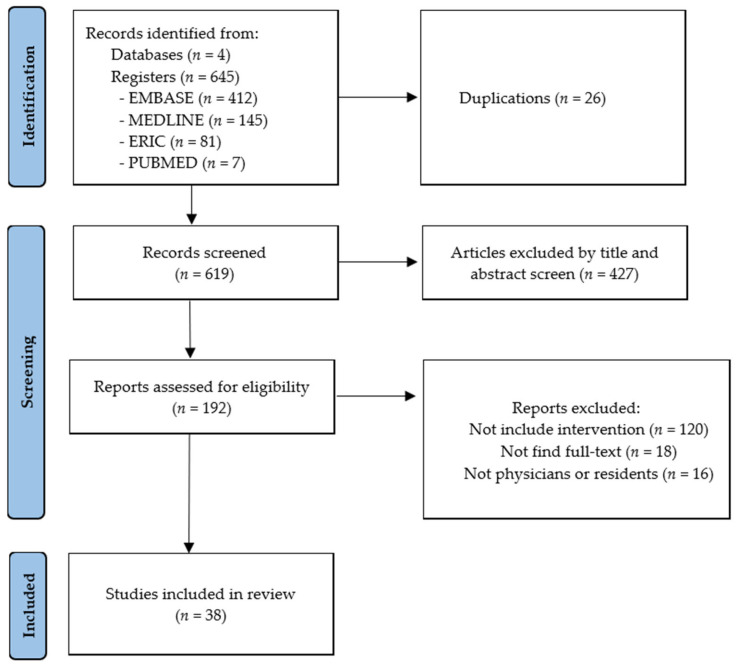
PRISMA 2020 flow diagram for scoping reviews which included searches of databases and registers.

This study employed a scoping review methodology to explore the literature on physician well-being strategies comprehensively. The scoping review was conducted by the Preferred Reporting Items for Systematic Reviews and Meta-Analyses (PRISMA) guidelines to ensure methodological rigour and transparency, and the procedure is illustrated in Figure 1. This study was retrospectively registered on the Open Science Framework (https://doi.org/10.17605/OSF.IO/8VN5W (accessed on 27 July 2025)).

### 2.2. Data Sources and Search Strategy

A comprehensive literature search was conducted using the following electronic databases: PubMed, Medline, Embase, and ERIC (Education Resources Information Centre).

The search strategy was developed in consultation with experts in the field and refined to ensure the inclusion of relevant studies. Boolean operators, Medical Subject Headings (MeSH) terms, and controlled vocabulary specific to each database were utilised to enhance the sensitivity and specificity of the search. The search was limited to peer-reviewed original research articles published in English. The queries used to search the four databases are presented in Appendix A. Medical Subject Heading terms and keywords related to the three portions of our question (well-being, physician, and medical education) were used to search these databases. Alternative terms for each terminology were used to ensure a thorough search. For example, well-being was regarded as a concept similar to ‘quality of life,’ ‘life satisfaction,’ and ‘coping,’ and these terms were also utilised in the literature search. Title and abstract screenings were conducted between 6 and 12 March 2025, and full-text reviews were conducted from 15 to 27 March 2025. Data extraction and synthesis occurred in April 2025.

### 2.3. Eligibility Criteria and Study Selection

Studies were selected based on predefined inclusion and exclusion criteria established by the research team. Eligible studies included those involving physicians, residents, and interns, focusing on well-being-related concepts such as stress, burnout, or mental health, conducted in clinical or healthcare training contexts, published in English, and employing qualitative, quantitative, or mixed-method study designs. No restrictions were placed on the publication date. The screening process was conducted in two phases: (1) Title and abstract screening and (2) Full-text review. Two independent reviewers carried out the selection process, and any discrepancies were resolved through discussion or consultation with a third reviewer. As illustrated in the PRISMA flow diagram in Figure 1, a total of 645 records were identified from four databases. After removing 26 duplicates, 619 records were screened by title and abstract, resulting in the exclusion of 427 records. A full-text review was conducted for the remaining 192 articles, of which 38 studies were included in the final analysis.

### 2.4. Data Extraction and Analysis

Relevant data from the included studies were extracted using a standardised data extraction form. Key information, including study characteristics, methodology, outcomes, and main findings, was synthesised to provide a comprehensive overview of the existing literature. The extracted data were analysed using a narrative synthesis approach, which involves the systematic organisation and textual description of findings from included studies to identify patterns, themes, and relationships relevant to the research question. This method was used to summarise and explain the similarities and differences in study findings, taking into account the methodological diversity and contextual variations [21].

The standardised data extraction form was designed to capture both general characteristics of the studies included in the analysis and specific attributes related to well-being strategies. The variables and coding criteria included in the data extraction form are summarised in Table 1. Descriptive variables extracted from each study included the publication year, PICOS framework components, methodology, medical education level, career level, geographic distribution, and physicians’ medical speciality. For studies such as surveys or interviews that did not involve an intervention, the intervention component of the PICOS framework was coded as “N (No).” The well-being strategy section included the names of the strategies used, their classification, and whether the study incorporated a positive deviance approach. If a study used both positive and negative strategies, the strategy classification was coded as “B (Both)”.

To analyse the strategies influencing physicians’ well-being, this study adopted an ecological systems framework, which enables a multi-level examination of individual, organisational, and socio-cultural influences. Originating from Bronfenbrenner’s ecological systems theory [20], this approach conceptualises human development and behaviour as shaped by nested and interacting environmental systems. In the context of medical practice, these include (1) the individual (e.g., characteristics of person, personality, and attitude), (2) the microsystem (e.g., interpersonal relationships and training programs), (3) the mesosystem (e.g., interactions between departments or roles), and (4) the macrosystem (e.g., institutional policies or administrative decisions, cultural norms, and national health policies). Applying this framework allows for a comprehensive understanding of well-being strategies that consider both proximal and distal determinants.

## 3. Results

### 3.1. Descriptives of the Included Studies for Physicians’ Well-Being Strategies

#### 3.1.1. Publication Year

The 38 papers selected for analysis in this scoping review were published between 2005 and 2024. The most recent year, 2024, accounted for the most significant number of publications, with eight studies (21.1%). In both 2023 and 2022, 5 studies were published (13.2% each), followed by four studies (10.5%) in 2018. A total of 2 studies (5.3%) were published in each of the following years: 2019, 2015, 2014, and 2013. One study (2.6%) was published in each of 2021, 2020, 2016, 2012, 2009, 2007, 2006, and 2005. No studies were published in 2008, 2010, 2011, or 2017. This distribution accounts for all 38 studies.

#### 3.1.2. Methodology

The 38 studies selected for analysis in this scoping review employed a range of methodological approaches. Of these, 27 studies (71.1%) used quantitative methods, 7 (18.4%) were qualitative, 3 (7.9%) adopted mixed-methods designs, and 1 study (2.6%) was descriptive.

#### 3.1.3. Medical Education and Career Level

The 38 studies selected for analysis in this scoping review addressed various stages of physicians’ education and training. Among them, 19 studies (50%) focused on physicians participating in continuing medical education (CME), while 17 studies (44.7%) targeted resident physicians in graduate medical education (GME). Two studies (5.3%) included overlapping groups, such as both residents and fellows or residents and faculty members.

Within this category, characteristics such as being female, having experienced trauma, or being in the early stages of a medical career were identified as relevant and distinct features related to the educational stage and career level.

#### 3.1.4. Geographic Distribution

The 38 studies selected for analysis in this scoping review were conducted across 12 countries. The United States accounted for the majority, with 25 studies (65.8%), followed by the United Kingdom with three studies (7.9%). One study (2.6%) was conducted in Australia, Canada, Germany, Indonesia, Iran, Israel, Norway, Spain, Switzerland, and Turkey.

#### 3.1.5. Physicians’ Medical Speciality

Of the 38 included studies, 13 specified the medical specialities of participating physicians. Among these 13 studies, both primary care and family medicine were represented in two studies each (15.4%). The remaining specialities—emergency medicine, general medicine, intensive care, internal medicine and surgery, obstetrics and gynaecology, otolaryngology, psychiatry, and surgery—were each represented in one study (7.7% each).

### 3.2. Physicians’ Well-Being Strategies

A typology of well-being strategies was developed based on educational and organisational levels, with each strategy further characterised by its deviance orientation—positive, negative, or both—as shown in Table 2.

Table 2 presents a summary of positive, negative, and deviant strategies identified across different educational levels and ecological systems in medical education. A total of 38 deviance strategies were identified, with the majority categorised as positive deviance (*n* = 26), followed by both deviance (*n* = 7) and negative deviance (*n* = 5). In the context of GME, 17 strategies were reported, of which 10 were positive deviance, two were negative deviance, and five were both types of deviance. Most GME strategies occurred at the microsystem (*n* = 13), with a predominance of positive deviance (*n* = 9) and smaller representations of negative (*n* = 2) and both deviance (*n* = 2). Other GME strategies were observed at the individual (*n* = 2) and multi-system (*n* = 2) levels. For CME, 19 strategies were identified, showing a strong skew toward positive deviance (*n* = 14), with three categorised as negative deviance and two categorised as both. Within CME, the majority of strategies were located at the microsystem level (*n* = 12), followed by individual (*n* = 3), macrosystems (*n* = 2), and multi-systems (*n* = 2). Positive deviance was especially prevalent in the microsystems (n = 10). A small number of strategies (*n* = 2) were applied to both graduate and continuing medical education, equally distributed at the individual and microsystem levels, and both were characterised as positive deviance. Overall, positive deviance strategies were most frequently reported at the microsystem (*n* = 20), highlighting the importance of localised team- or unit-level interventions in both GME and CME settings.

**Figure 2:**
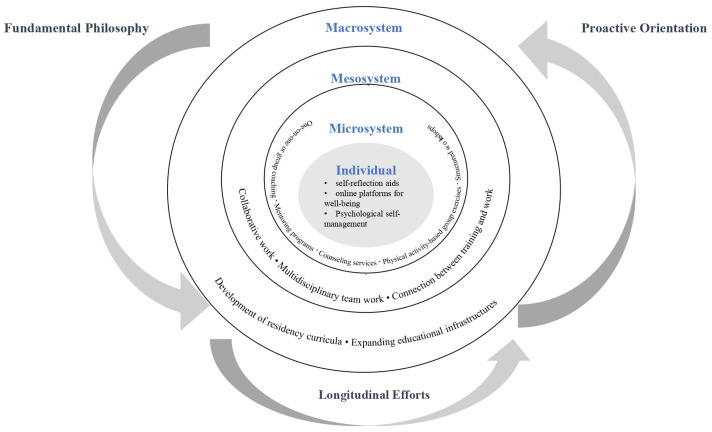
Ecological system of positive deviance strategies for physicians’ and residents’ well-being.

Strategies at the individual level involve utilising personal tools and resources—such as self-reflection aids, online platforms, and other supportive instruments—to monitor and safeguard one’s psychological well-being. Microsystem-level strategies encompass interventions such as one-on-one or group coaching, mentoring programs, physical activity-based group exercises, counselling services, and structured workshops. Studies aiming to transform organisational and educational culture—through initiatives such as the development of residency curricula or the establishment of educational infrastructures like libraries—are categorised at the macrosystem level. Additionally, several studies adopt multi-level approaches, implementing strategies that span across individual, microsystem, and macrosystem domains. Figure 2 illustrates a diagram categorising examples of positive deviance strategies by ecological system levels. These include comprehensive interventions such as revising hospital-wide guidelines, promoting organisational culture change, reforming supervisory practices in residency education, and concurrently supporting individual-level well-being initiatives.

### 3.3. Characteristics of Positive Deviance Strategies

A total of 26 studies that applied positive deviance strategies were examined in this scoping review. The synthesis revealed four overarching characteristics: an underlying philosophy that promotes effectiveness and professional values, a proactive approach to physician well-being, the diffusion of positive behaviours, and the implementation of longitudinal efforts to sustain effective practices. The three characteristics derived from the review results are shown in Figure 2.

#### 3.3.1. Foundational Philosophy of Enhancing Professional Values

Positive deviance strategies are often grounded in a comprehensive philosophy that prioritises the reinforcement of core professional values, such as empathy, authenticity, and patient-centredness. These strategies do not merely aim to alleviate distress or reduce burnout; instead, they seek to elevate professional identity and foster moral engagement among physicians.

For instance, Study 14 in Appendix B regarded physician well-being as a fundamental value and implemented organisational initiatives to enhance the workplace culture. This study recognised that the culture surrounding physicians significantly influences their well-being and that the well-being of all healthcare professionals enhances both patient-centred care and the overall meaning of medical practice. Notably, Study 38 found that effective time management by physicians impacts not only their performance but also their well-being, emphasising the importance of productivity, efficiency, and sustainability.

In summary, the pursuit of physician well-being was not treated as a separate agenda but as an essential component in realising the mission and vision of healthcare practice. It was intrinsically linked to the value of enabling physicians to fulfil meaningful and professionally significant roles.

#### 3.3.2. Proactive Orientation

A defining characteristic of positive deviance strategies was their proactive nature. These interventions did not merely address burnout or stress-related outcomes retrospectively; instead, they focused on anticipating potential challenges to physician well-being and implementing preventive measures to address them. This approach likely reflects insights gained from longitudinal survey studies and interview-based research that have identified predictive factors negatively influencing well-being, job satisfaction, and quality of life across various medical specialities and training levels.

For instance, Study 36 in Appendix B implemented a modified mindfulness-based stress reduction program for first-year surgical residents who face significant emotional, psychological, and cognitive challenges during their initial training year. The intervention consisted of weekly 2 h in-person sessions complemented by 20 min daily home practices over eight weeks, with outcomes tracked for one year. Results demonstrated stabilised performance in emotion regulation tasks, indicating enhanced well-being and clinical competency among participants.

These findings suggest that positive deviance strategies operate through a proactive orientation, cultivating resilience and psychological stability via structured curricula and training programs before stress or burnout manifests. This contrasts with traditional reactive approaches, which focus on mitigating symptoms after they have emerged. The emphasis on preemptive skill-building—particularly in high-stakes specialities like surgery—highlights the importance of integrating well-being frameworks into medical education and workplace systems.

#### 3.3.3. Diffusion of Positive Behaviours

Several studies highlighted that positive behaviours within clinical and educational settings tend to diffuse subtly and often without explicit recognition. Rather than relying on large-scale campaigns, conspicuous activities, or one-off training sessions, positive deviance strategies focus on organic, quiet interventions—such as mobile meditation and coaching—that gradually foster habitual and routine behaviours over time.

For example, Study 5 in Appendix B conducted in-depth interviews to understand the physical, social, and psychological impacts of COVID-19 on physicians. The findings underscored the importance of changes in work environments and systems, emphasising that physicians’ everyday surroundings significantly influence their well-being and that strengthening both psychological and social support is essential. Similarly, Study 38 stressed the importance of integrating well-being curricula into residency and fellowship programs to support the well-being of residents and fellows, reporting preliminary results from the development and implementation of such a curriculum.

These findings suggest that the effectiveness of positive deviance strategies lies not in formal implementation or incentives, but in broad changes to the everyday environment and curriculum improvements. Ultimately, the diffusion of positive behaviours is achieved through incremental, contextually embedded changes that become part of daily practice, rather than through top-down directives or isolated interventions.

#### 3.3.4. Good Practice as a Longitudinal Effort

The results of this scoping review indicate that strategies to enhance physician well-being have been implemented over a wide range of durations, from as brief as 45 min to as long as one year. Notably, most positive deviance strategies were applied over periods ranging from several days to several months. In particular, interventions such as coaching, mentoring, and training programs focused on reflection or mindfulness were typically conducted for eight weeks or longer.

For example, Study 18 in Appendix B emphasised the significant impact of physicians’ psychological states on the quality of patient care. To address this, the study implemented an eight-week mindfulness training program and monitored participants’ relaxation states over a year. The findings demonstrated that the intervention group, compared to the control group, experienced enhanced relaxation, improved perceptions of well-being, and greater awareness of their health. The primary objective of this study was to enhance the efficiency of economic and social resources by improving the quality of patient care through physician well-being. Similarly, Study 17 employed an online training program over three months and observed positive changes in work and job satisfaction among participants.

These findings suggest that longitudinal strategies are necessary to sustain physicians’ stable mental states and to facilitate organisational or cultural change. The characteristics of effective interventions included continuous engagement, ongoing feedback, and repeated opportunities for reflection and improvement. This temporal dimension underscores the importance of internalising well-being practices among physicians for lasting impact.

## 4. Discussion

This study aims to examine the research landscape concerning positive deviance strategies intended to promote physicians’ well-being and quality of life. Additionally, it examines the literature on positive and negative deviance strategies. Through the lens of positive deviance strategies, the review aims to generate meaningful implications for physicians’ well-being. Based on the analysis results, several points were discussed. First, it highlights the increasing diversity and evolution of methodologies used in recent research on physician well-being strategies. Second, it underscores the importance of applying an ecological systems perspective, noting that positive deviance strategies at the microsystem level are particularly effective. Third, it discusses the foundational philosophy, proactive orientation, diffusion of positive behaviours, and the necessity of sustained, long-term efforts as core implications of positive deviance strategies for enhancing physician well-being.

### 4.1. Methodology for the Studies of Physicians’ Well-Being Strategies

Recent research on strategies to promote physician well-being has increasingly adopted a variety of methodological approaches, including qualitative, quantitative, and mixed-methods designs. Mixed-methods research, in particular, has gained prominence for its ability to capture the complexity of physician well-being by integrating numerical data with in-depth qualitative insights [19,20,21,22]. This methodological diversity reflects the recognition that physician well-being is a multidimensional construct influenced by personal, organisational, and systemic factors.

The COVID-19 pandemic has catalysed an expansion of research in this area, leading to a surge in studies that address not only burnout and psychological distress but also resilience, coping mechanisms, and systemic challenges faced by physicians [23,24,25]. For example, recent bibliometric analyses have documented a significant increase in publications on physician mental health since 2020, with dominant themes including burnout, depression, and anxiety [26]. These studies have also highlighted the need for standardised assessment tools and the importance of addressing systemic contributors, such as excessive workload, hierarchical structures, and inadequate institutional support.

Research conducted across diverse geographic regions, including North America, Europe, and Asia, demonstrates that physician well-being is a global concern; however, disparities in research output and intervention implementation persist between high-income and low- and middle-income countries [27,28,29,30]. Furthermore, studies have underscored the importance of considering individual characteristics—such as training level, career stage, gender, and personal experiences (including trauma)—when developing and evaluating well-being interventions [20,31]. For instance, qualitative and mixed-methods studies have revealed that factors such as hope, social intelligence, and teamwork are variably associated with well-being outcomes, depending on the physician’s context and personal attributes [20].

Given these complexities, it is essential for future research to employ integrative and context-sensitive methodologies. Such approaches should combine quantitative measures (e.g., validated well-being indices) with qualitative data (e.g., interviews, focus groups) to capture nuanced experiences [19,22]. The approaches also address both individual and organisational determinants of well-being, recognising their dynamic interplay [30,32]. The approaches propose and evaluate tailored interventions that account for specific contextual and personal factors, ensuring relevance and effectiveness across diverse settings [31,33].

By advancing methodological rigour and inclusivity, the field can better inform evidence-based, individualised, and system-level strategies to enhance physician well-being worldwide.

### 4.2. Comprehensive Lens for Ecological System of Physicians’ Well-Being Strategies

The ecological systems theory provides a comprehensive and dynamic framework for examining the multilayered strategies aimed at improving physicians’ professional well-being. By accounting for interactions across multiple environmental levels—ranging from the individual to the macrosystem—this model highlights the importance of contextual and developmental sensitivity in designing and implementing well-being interventions [11,34,35]. Within this framework, the predominance of positive deviance strategies at the microsystem level emerges as a particularly salient trend. This reflects the effectiveness of locally embedded, interpersonal, and team-based interventions, such as peer support groups, coaching, and collaborative problem-solving, in fostering sustainable improvements in well-being [36,37].

Microsystem-level strategies are inherently more adaptable to diverse clinical and educational environments, requiring fewer institutional resources while leveraging relational dynamics and peer influence. Their suitability for both Graduate Medical Education (GME) and Continuing Medical Education (CME) contexts underscores their utility in addressing the immediate needs of physicians at different career stages [38]. Furthermore, the emphasis on positive deviance represents a paradigm shift from deficit-focused approaches toward identifying and amplifying successful behaviours already present within healthcare systems [19,34]. By celebrating what works well under challenging conditions, positive deviance strategies foster a culture of resilience, collective efficacy, and innovation, promoting both individual and organisational flourishing.

The emergence of strategies categorised as “both deviance”—which integrate elements of both corrective action and exemplary modelling—further highlights the complexity of physician well-being initiatives. These hybrid approaches offer a balanced framework that simultaneously addresses existing deficits while promoting aspirational practices, thereby enhancing both accountability and engagement across stakeholders [38]. The comparatively lower frequency of negative deviance strategies may reflect a broader conceptual shift: although reactive interventions remain necessary in acute situations, they are increasingly viewed as insufficient for achieving long-term, system-wide transformation [9].

In sum, the ecological orientation and positive deviance paradigm jointly advocate for a strengths-based, contextually grounded approach to physician well-being. Particularly at the microsystem level, such strategies are well-positioned to empower individuals, strengthen professional identity, and create conditions conducive to sustained engagement and well-being. Future research and policy should prioritise the integration of these approaches into medical training and workplace systems, promoting resilient healthcare environments grounded in evidence-based and human-centred principles.

### 4.3. Implications of Physicians’ Well-Being Strategies

A comprehensive review of the literature on positive deviance strategies for physician well-being reveals four central implications, each supported by empirical and theoretical research.

Positive deviance strategies are deeply rooted in a philosophy that prioritises the enhancement of core professional values, such as empathy, authenticity, and patient-centredness. These strategies do not merely aim to reduce distress or burnout; instead, they seek to elevate professional identity and foster moral engagement among physicians. This value-driven orientation is echoed in studies demonstrating that positive deviance can reinforce organisational values and contribute to a culture of safety and excellence in healthcare settings [17,18,19].

Unlike traditional, reactive interventions that address stress or burnout after they occur, positive deviance strategies emphasise a proactive stance. They focus on anticipating challenges to physician well-being and implementing preventive measures, such as resilience training and mindfulness programs, before problems manifest. This proactive orientation has been shown to enhance psychological stability and clinical competency, supporting the long-term well-being of healthcare professionals [17,39].

A key strength of the positive deviance approach is its ability to facilitate the organic diffusion of effective behaviours within clinical and educational settings. Rather than relying on top-down mandates or isolated interventions, positive deviance leverages the influence of exemplary individuals or teams whose successful practices are gradually embedded in the broader organisational culture. This process of social proof and peer learning has been documented as a powerful mechanism for spreading best practices and improving care quality [18,20,30].

Sustained, longitudinal engagement is essential for the successful implementation of positive deviance strategies. Research indicates that interventions such as coaching, mentoring, and reflective training are most effective when delivered over extended periods, allowing for the internalisation of new behaviours and the gradual transformation of organisational norms. Long-term programs grounded in positive deviance principles have been associated with improved well-being, reduced burnout, and enhanced patient outcomes [20,35].

Collectively, these findings underscore the transformative potential of positive deviance as a framework for physician well-being. By promoting the autonomous and intrinsically motivated spread of positive behaviours, positive deviance not only strengthens individual resilience but also fosters a culture of continuous improvement and professional fulfilment. This approach moves beyond temporary stress management, instead supporting the restoration and reinforcement of essential values and the cultivation of a supportive organisational environment [19,20,39].

To establish a sustainable environment for physician well-being, healthcare institutions should prioritise the development and implementation of strategically planned, long-term programs that are firmly grounded in the principles of positive deviance. Such efforts are likely to yield more enduring and meaningful improvements in both professional satisfaction and the quality of patient care.

### 4.4. Practical and Policy Implications

The findings of this review have important implications for both practice and policy in healthcare settings. While positive deviance strategies show promise for enhancing physician well-being, their successful translation into practice requires careful consideration of several real-world factors.

First, adopting positive deviance approaches in healthcare is often hindered by barriers such as limited resources, resistance to change, and variability in organisational culture [22]. Effective implementation demands not only the identification of successful practices but also strategies to overcome these obstacles, including engaging frontline staff, fostering leadership support, and ensuring that interventions are feasible within existing resource constraints [23].

Second, the dynamics within healthcare institutions—such as leadership styles, communication patterns, and interdepartmental collaboration—play a critical role in the adoption and sustainability of positive deviance strategies [23,40,41]. Institutions that encourage openness, shared learning, and the dissemination of best practices are more likely to see successful integration of these approaches. Conversely, hierarchical or siloed environments may impede the spread of positive behaviours.

Third, for positive deviance to have a broad and lasting impact, system-level strategies are essential [19,34]. These include establishing supportive policy frameworks, integrating positive deviance principles into quality improvement initiatives, and creating mechanisms for cross-organisational learning. Systematic identification of high-performing units, rigorous evaluation of their practices, and structured dissemination of findings can facilitate the scaling of effective interventions across healthcare systems.

By addressing these practical, institutional, and systemic factors, healthcare organisations can more effectively leverage positive deviance strategies to promote resilience, well-being, and sustained improvement in clinical practice. Future research and policy should prioritise the development of implementation guidelines and the evaluation of system-level interventions to maximise the real-world impact of positive deviance in healthcare.

### 4.5. Strengths, Limitations, and Future Directions

This study offers a comprehensive synthesis of positive deviance strategies for enhancing physician well-being, mapping a wide range of interventions across educational and ecological system levels. By reviewing both positive and negative deviance approaches, the study provides valuable insights into the diversity and characteristics of strategies currently employed in medical education and practice. The use of a scoping review methodology, adherence to PRISMA guidelines, and the inclusion of studies from multiple countries further strengthen the credibility and breadth of the findings.

However, several limitations should be considered when interpreting these results. First, the review focused on cataloguing the range of strategies rather than evaluating their effectiveness; thus, the comparative impact of different interventions remains unclear. Future research, including meta-analyses, is needed to determine which strategies are most effective in promoting physician well-being. Second, the analysis did not differentiate between medical specialities or account for the influence of accreditation standards such as those set by the ACGME. As a result, the findings may not fully capture speciality-specific needs or regulatory contexts. Third, the literature search was limited to English-language publications, which may have led to the exclusion of relevant studies in other languages and introduced potential language bias. Fourth, despite employing a comprehensive set of keywords and MeSH terms, some relevant studies may have been missed due to limitations in database indexing or search strategy. Fifth, although study selection and data extraction were conducted independently by two reviewers, the possibility of selection bias remains due to subjective judgement in the screening process.

To address these limitations, future studies should aim to evaluate the effectiveness of specific positive deviance strategies through rigorous comparative research and meta-analyses. Expanding the scope to include non-English literature and speciality-specific analyses will help ensure a more comprehensive understanding of physician well-being interventions. Additionally, research that considers the impact of accreditation standards and explores strategies tailored to different clinical contexts will further enhance the applicability of findings. Continued efforts to refine search methodologies and minimise reviewer bias will also strengthen the quality of future reviews.

## 5. Conclusions

This scoping review provides a comprehensive synthesis of positive deviance strategies aimed at promoting physician well-being across educational and ecological system levels. The findings highlight that positive deviance strategies—particularly those implemented at the microsystem level—are characterised by a foundational commitment to professional values, a proactive orientation, the subtle diffusion of positive behaviours, and sustained, longitudinal engagement. These approaches not only address individual and organisational factors but also foster resilience, professional identity, and psychological safety among physicians. By shifting the focus from deficit-based interventions to the amplification of effective, context-sensitive practices already present within healthcare environments, positive deviance offers a promising and sustainable framework for advancing physician well-being. Future research and institutional efforts should prioritise the integration of positive deviance principles into well-being initiatives, ensuring that strategies are both adaptable and embedded within daily clinical practice. Ultimately, cultivating a culture of well-being through positive deviance has the potential to enhance not only the quality of physicians’ professional lives but also the overall quality of patient care.

Nevertheless, this study is significant in that it provides a comprehensive overview of the diverse strategies currently employed to enhance physician well-being. The findings offer a foundation for the development and application of more effective and sustainable positive deviance strategies in the future.

## Figures and Tables

**Table 1:** Data Extraction Variables and Coding Criteria.

Category	Variables	Coding
Descriptive	Publication year	Year
	PICOS *	Yes/No
	Methodology	Quantitative/Qualitative/Mixed/Descriptive
	Medical Education Level	GME/CME/Both
	Career level	Faculty/Resident/Intern
	Geographic distribution	Country name
	Medical specialty	Specialty name
Well-being strategy	Classification of strategy	Positive/Negative/Both
	Ecological system	Individual/Microsystem/Mesosystem/Macrosystem

* PICOS: Population, Intervention, Comparison, Outcomes, and Study Design.

**Table 2:** Positive and Negative Deviance Strategies.

Educational Level	Positive Deviance	Negative Deviance	Both Deviance	Total
Graduate Medical Education (GME)	10	2	5	17
Individual			2	2
Microsystem	9	2	2	13
Multi-system	1		1	2
Continuing Medical Education (CME)	14	3	2	19
Individual	1	1	1	3
Microsystem	10	1	1	12
Macrosystem	1	1		2
Multi-system	2			2
Graduate and Continuing Medical Education	2			2
Individual	1			1
Microsystem	1			1
Total	26	5	7	38

## Data Availability

The data utilised in this review are provided in the Appendix A and Appendix B.

## Abbreviations

The following abbreviations are used in this manuscript:

GME	Graduate Medical Education
CME	Continuing Medical Education
ACGME	Accredited Council of Graduate Medical Education

## Appendix A

The search terms utilized in this study are presented below.

PubMed Central

((well-being[Title] OR quality of life[Title] OR life satisfaction[Title] OR coping[Title]) AND (physician[Title] OR doctor[Title] OR internship[Title] OR residency[Title] OR faculty[Title])) OR (((well-being[Abstract] OR quality of life[Abstract] OR life satisfaction[Abstract] OR coping))[Abstract] AND (physician[Abstract] OR doctor[Abstract] OR internship[Abstract] OR residency[Abstract] OR faculty[Abstract])) AND (medical education[Abstract] OR graduate medical education[Abstract] OR GME[Abstract] OR continuing medical education[Abstract] OR CME[Abstract])

ERIC

TI ((well-being or wellbeing or well being or quality of life OR life satisfaction OR coping) AND (physician or doctor or internship or residency or faculty)) OR AB ((well-being or wellbeing or well being or quality of life OR life satisfaction OR coping) AND (physician or doctor or internship or residency or faculty)) AND AB (Medical education OR Graduate Medical education OR GME OR Continuing Medical Education OR CME)

APA PsychInfo

title((well-being OR quality of life OR life satisfaction OR coping) AND (physician OR doctor OR internship OR residency OR faculty)) OR abstract((well-being OR quality of life OR life satisfaction OR coping) AND (physician OR doctor OR internship OR residency OR faculty)) AND abstract(medical education OR graduate medical education OR GME OR continuing medical education OR CME)

EMBASE

(‘wellbeing’ OR ‘quality of life’ OR ‘life satisfaction’ OR ‘coping’):ab,ti AND ((‘physician’ OR doctor OR ‘internship’ OR ‘residency’ OR faculty):ab,ti) AND ((‘medical education’ OR ‘graduate medical education’ OR GME OR ‘continuing medical education’ OR CME):ab)

Medline

((well-being[Title] OR “quality of life”[Title] OR “life satisfaction”[Title] OR coping[Title]) AND (physician[Title] OR doctor[Title] OR internship[Title] OR residency[Title] OR faculty[Title])) OR ((well-being[Title/Abstract] OR “quality of life”[Title/Abstract] OR “life satisfaction”[Title/Abstract] OR coping[Title/Abstract]) AND (physician[Title/Abstract] OR doctor[Title/Abstract] OR internship[Title/Abstract] OR residency[Title/Abstract] OR faculty[Title/Abstract]) AND (“medical education”[Title/Abstract] OR “graduate medical education”[Title/Abstract] OR GME[Title/Abstract] OR “continuing medical education”[Title/Abstract] OR CME[Title/Abstract])) AND medline[sb].

## Appendix B

The list of 38 academic papers reviewed in this study is as follows.

**Table A1:** Overview of the 38 academic papers included in this study.

No.	Year	Title	Educational Level	Methodology	Country	Strategy	Ecological System	Positive/Negative
1	2024	Implementing an interactive self-screening psychological distress tool for residents and fellows [42]	GME	Quantitative	US	Confidential, Online, Interactive screening program(ISP)	Micro	N
2	2024	Medical doctors’ coping strategies with post-earthquake stress and their relationship with presenteeism [43]	CME	Quantitative	Turkey	Coping strategies: Positive re-evaluation	Individual	P
3	2024	The protective role of sense of coherence in resident physicians facing secondary trauma due to patient death in intensive care—a qualitative inquiry [44]	GME	Qualitative	Israel	Salutogenic-based interventions	Macro/Micro/Individual	P
4	2024	Evaluation of integrative medicine in residency-psychiatry curriculum [45]	GME	Mixed	US	Residency-Psychiatry (IMR-Psychiatry) curriculum, A 100-h elective	Macro	P
5	2024	Perceived implications of COVID-19 on physical, mental and social aspects of resident doctors in a tertiary care hospital [46]	GME	Qualitative	Canada	Mental aid, Social support, Ample resources and decreasing their work burden	Individual	B
6	2024	Psychological experiences and reactions of physicians with COVID-19: A qualitative study [47]	CME	Qualitative	Iran	Adaptive/Maladaptive Copings	Individual	B
7	2023	Association between hours of work and subjective well-being. How do physicians compare to lawyers and accountants? [48]	CME	Quantitative	UK	Work hours	Macro	N
8	2023	Implementation and effectiveness of a physician-focused peer support program [49]	CME	Quantitative	UK	Peer Outreach Support Team (POST) program	Micro	P
9	2023	Underutilization of effective coping styles in male physicians with burnout [50]	CME	Quantitative	Switzerland	Coping strategies	Individual	N
10	2023	Burnout and coping strategies among resident physicians at an Indonesian tertiary referral hospital during COVID-19 pandemic [51]	GME	Quantitative	Indonesia	Adaptive/Maladaptive Copings	Individual	B
11	2022	The effects of a tailored mindfulness-based program on the positive mental health of resident physicians—A randomized controlled trial [52]	GME	Quantitative	Germany	Mindfulness-based program (MBP)	Micro	P
12	2022	Effectiveness of a multi-modal hospital-wide doctor mental health and wellness intervention [53]	CME	Quantitative	Australia	Multimodal intervention	Macro	P
13	2020	Coaching for primary care physician well-being: A randomized trial and follow-up analysis [54]	CME	Quantitative	US	Positive psychology-based coaching	Micro	B
14	2019	Monitoring the organizational wellness of a physician residency program: A quality improvement process [55]	GME	Mixed	US	Wellness 2.0	Macro/Individual	B
15	2018	Positive value of a women’s junior faculty mentoring program: A mentor-mentee analysis [56]	CME	Quantitative	US	A mentoring program for women junior faculty	Micro	P
16	2018	Emotional distress among physician residents and fellows: An observational study of trainees seeking counseling visits [57]	GME	Qualitative	US	On-site counseling services	Micro	P
17	2016	A randomized, controlled study of an online intervention to promote job satisfaction and well-being among physicians [58]	CME	Qualitative	US	Individualized, Online intervention	Micro	P
18	2015	Learning to see beneath the surface: A qualitative analysis of family medicine residents’ reflections about communication [59]	GME	Qualitative	US	Reflective exercises program	Micro	P
19	2015	Enhancing relaxation states and positive emotions in physicians through a mindfulness training program: A one-year study [60]	CME	Quantitative	Spain	Mindfulness-based stress reduction (MBSR)	Micro	P
20	2014	Engaging residents and fellows to improve institution-wide quality: The first six years of a novel financial incentive program [61]	GME, CME	Quantitative	US	Financial incentives program	Micro	P
21	2014	Increasing physician activity with treadmill desks [62]	CME	Quantitative	US	Treadmill + Device + Counseling	Micro	P
22	2013	Transitions in medicine: Trainee doctor stress and support mechanisms [63]	CME	Mixed	UK	Lessen anxiety and assist transition	Macro/Individual	P
23	2012	A mindfulness course decreases burnout and improves well-being among healthcare providers [64]	CME	Quantitative	US	Education course based on mindfulness-based stress reduction	Micro	P
24	2009	Association of an educational program in mindful communication with burnout, empathy, and attitudes among primary care physicians [65]	CME	Quantitative	US	Intensive educational program	Micro	P
25	2007	Meeting the imperative to improve physician well-being: Assessment of an innovative program [66]	CME	Quantitative	US	Physician control over the work environment, Order in the clinical setting, and Clinical meaning	Macro/Micro	P
26	2006	Combating the Stress of Residency: One School’s Approach [67]	GME	Descriptive	US	Residency Assistance Program (RAP)	Micro	P
27	2005	Professional secrecy as a stressor among Norwegian physicians [68]	CME	Quantitative	Norway	MM (mortality and morbidity)-meetings	Micro	P
28	2024	Impact of a Participatory Wellness Continuing Medical Education Program on Physician Burnout and Well-Being [69]	CME	Quantitative	US	A 3-day wellness CME program.	Micro	P
29	2023	Online Well-Being Group Coaching Program for Women Physician Trainees: A Randomized Clinical Trial [70]	CME	Quantitative	US	A 4-month, Web-based, Group coaching program(BetterTogetherPhysician Coaching)	Micro	N
30	2022	Financial Literacy and Physician Wellness: Can a Financial Curriculum Improve an Obstetrician/Gynecologist Resident and Fellow’s Well-Being? [71]	GME	Quantitative	US	A five-part personal financial literacy curriculum	Micro	P
31	2013	Physical activity, quality of life, and burnout among physician trainees: The effect of a team-based, incentivized exercise program [72]	GME	Quantitative	US	An elective, team-based, 12-week, incentivized exercise program	Micro	B
32	2018	Implementing a Narrative Medicine Curriculum During the Internship Year: An Internal Medicine Residency Program Experience [73]	GME	Qualitative	US	Narrative medicine session	Micro	P
33	2022	Effect of a Novel Online Group-Coaching Program to Reduce Burnout in Female Resident Physicians: A Randomized Clinical Trial [74]	GME	Quantitative	US	A structured professional group-coaching program	Micro	N
34	2024	Physician Coaching by Professionally Trained Peers for Burnout and Well-Being: A Randomized Clinical Trial [75]	CME	Quantitative	US	Coaching sessions facilitated by a peer coach	Micro	P
35	2022	Mobile meditation for improving quality of life, anxiety and depression among surgical residents and faculty [76]	GME, CME	Quantitative	US	Mobile meditation	Individual	P
36	2019	Efficacy of Mindfulness-Based Cognitive Training in Surgery: Additional Analysis of the Mindful Surgeon Pilot Randomized Clinical Trial [77]	GME	Quantitative	US	Mindfulness-based stress reduction (modMBSR) class	Micro	P
37	2018	Physician Time Management [78]	GME	Quantitative	US	Effective time-management strategies	Micro	P
38	2021	Well-being curriculum for anesthesiology residents: Development, processes, and preliminary outcomes [79]	GME	Quantitative	US	Well-being curriculum	Micro	B
